# Evaluation of Improvest effects on production parameters of gilts from two different genetic sire lines

**DOI:** 10.1093/tas/txad144

**Published:** 2023-12-23

**Authors:** Manuel A Vasquez-Hidalgo, Martha Mellencamp, Deborah Amodie, Benjamin M Bohrer, Leanne VanDeWeyer, Kimberly A Vonnahme

**Affiliations:** Department of Animal Sciences, North Dakota State University, Fargo, ND 58108-6050, USA; Zoetis, Inc., Parsippany, NJ 07054, USA; Zoetis, Inc., Parsippany, NJ 07054, USA; Department of Animal Sciences, The Ohio State University, Columbus, OH 43210, USA; Zoetis, Inc., Parsippany, NJ 07054, USA; Zoetis, Inc., Parsippany, NJ 07054, USA

**Keywords:** Improvest, growth performance, gilts, barrows, carcass

## Abstract

The objective was to evaluate the effects of Improvest on the performance and carcass characteristics of gilts from two different genetic sire lines and the performance of Improvest gilts with castrated male pigs. It was hypothesized that performance parameters observed for Improvest gilts would be similar to barrows, thus narrowing the performance gaps between traditionally managed gilts and barrows. Pigs were from Large White/Landrace dams and either Duroc–Pietrain (**DP**) or Duroc (**D**) sires. Females within each genetic sire line were randomized by weight to receive the first dose of Improvest (**IMP**) on day 25 of the study or to serve as a nontreated control (DP IMP gilt (*n* = 6 pens; 19 pigs/pen), D IMP gilt (*n* = 6 pens; 19 pigs/pen), DP CON gilt (*n* = 6 pens; 19 pigs/pen), D CON gilt (*n* = 6 pens; 19 pigs/pen). The second dose of Improvest was administered 6 wk later (i.e., day 67). Barrows did not receive Improvest (DP barrow [*n* = 10 pens; 19 to 20 pigs/pen], D barrow [*n* = 10 pens; 19 to 20 pigs/pen]). Average daily gain (**ADG**), average daily feed intake (**ADFI**), and gain-to-feed ratio (**G:F**) were measured at 21 d intervals throughout the duration of the study. The targeted weight for pigs to be marketed was 133 ± 2.5 kg. Carcass characteristics and loin quality parameters were evaluated on a subset of pigs (*n* = 283). Improvest-treated gilts of both genetic lines had increased (*P* ≤ 0.05) ADG and ADFI compared to untreated gilts during the post-second dose intervals with values exceeding that of barrows from day 84 to marketing. Overall, DP IMP gilts had increased (*P* ≤ 0.05) G:F post-second dose compared to DP CON gilts and DP barrows, yet all other treatments were similar. As pigs were marketed at a similar weight, there was no difference in the final weight, however, DP IMP gilts and DP barrows reached market weight sooner (*P* ≤ 0.05) than DP CON gilts (109.9 and 111.8 vs. 114.3 ± 0.8 d). Backfat and loin weight were greater (*P* < 0.01) in IMP gilts versus CON gilts, while IMP gilts and barrows had similar values within each respective genetic sire line. There were no differences between treatments (*P* ≥ 0.08) for pH and instrumental color of the loins. When the pass rate of loins (Japanese color score ≥ 3.0 and marbling ≥ 2.0) was evaluated, IMP gilts were at intermediate values between CON gilts and barrows for each respective genetic sire line. Overall, Improvest within a genetic line improved gilt carcass measurements so that they were more similar to barrows.

## Introduction

There is a well-known gap between gilt and barrow production performance and carcass characteristics. Gilts have a decreased average daily gain (**ADG**), average daily feed intake (**ADFI**), backfat, and marbling compared to barrows (Woodworth et al., 2021). Gonadotropin-releasing factor analog-diphtheria toxoid conjugate (i.e., Improvest) improves gilt performance and carcass characteristics by increasing ADG, ADFI, and backfat compared to gilts not receiving Improvest (Oliver et al., 2003; Rodriguez et al., 2019; Nautrup et al., 2020; Allison et al., 2021; Pérez-Ciria et al., 2021; Vasquez-Hidalgo et al., 2023).

Improvest works through the production of antibodies against gonadotropin-releasing factor (GnRF; Dunshea et al., 2001; Bradford and Mellencamp, 2013). These antibodies hinder the production of GnRF after a second dose of Improvest was administered. With a lack of GnRF, the stimulation of the anterior pituitary for the production of follicular stimulating hormone and luteinizing hormone is prevented, resulting in decreased steroidal production by the gonads (Dunshea et al., 2001; Pérez-Ciria et al., 2021). Without sexual hormones, estrous behavior is absent in the gilt. Total uterine and ovarian weights are decreased in Improvest-treated gilts (Rodriguez et al., 2019; Pérez-Ciria et al., 2021). Similarly, ovarian follicle maturation is severely hindered in Improvest-treated gilts (Pérez-Ciria et al., 2021).

Physically ovariectomized gilts have shown improvement in weight gain and fat deposition when compared to cycling gilts (Oliver et al., 2003; Van den Broeke et al., 2016). Improvest increases gilt growth and carcass fat deposition approximating their development to that of barrows (Bohrer et al., 2014; Nautrup et al., 2020), yet direct Improvest gilt versus barrow comparisons are limited in peer-reviewed literature. Moreover, estrus in gilts is known to suppress feed intake (Almeida et al., 2000) and cause behavioral problems (i.e., mounting, aggression), therefore treated gilts experience an overall increase in feed intake after the second dose of Improvest is administered (Rodrigues et al., 2019; Nautrup et al., 2020; Vasquez-Hidalgo et al., 2023). Studies in gilts have shown that Improvest increases ADG and ADFI post-second dose with varying effects in gain-to-feed ratio (**G:F**; Oliver et al., 2003; Bohrer et al., 2014; Van den Broeke et al., 2016; Rodriguez et al., 2019; Nautrup et al., 2020; Allison et al., 2021; Pérez-Ciria et al., 2021; Vasquez-Hidalgo et al., 2023).

Pietrain-sired terminal pigs are generally used because of an advantage in certain carcass characteristics such as lean percentage and muscularity (Augspurger et al., 2002; Edwards et al., 2006; Morales et al., 2013). Nevertheless, performance characteristics, backfat deposition, and meat quality traits related to fat deposition (i.e., marbling and belly thickness) are not optimal compared to pigs sired from other breeds (Augspurger et al., 2002; Edwards et al., 2006; Morales et al., 2013). Duroc-sired pigs have increased performance characteristics such as ADG and ADFI compared to pigs sired from other breeds such as Landrace, Large White, and Pietrain (McGloughlin et al., 1988; Augspurger et al., 2002; Edwards et al., 2006). Pietrain-sired barrows show decreased ADG, ADFI, and similar feed efficiency compared with Duroc-sired barrows (Morales et al., 2013). However, Improvest-treated Pietrain-sired barrows show greater ADG and feed efficiency post-second dose compared to surgically castrated Duroc-sired barrows (Morales et al., 2013). This along with an increase in ADFI in Improvest-treated Pietrain-sired barrows resulted in similar performance characteristics between Pietrain and Duroc-sired barrows (Morales et al., 2013). To our knowledge, there are no studies investigating the effect of Improvest in the performance and carcass characteristics of Duroc- and Pietrain-sired gilts.

The objective of this study was to confirm the positive effects of Improvest for performance and carcass characteristics in gilts in two different genetic lines of pigs (i.e., Duroc–Pietrain (**DP**)-sired or Duroc-sired). We also investigated if the use of Improvest could lead to greater weight uniformity in an established market weight scenario by decreasing the performance gaps typically observed between market gilts and castrated barrows. Our hypothesis was that Improvest would increase ADG, ADFI, and maintain feed efficiency in treated gilts compared to untreated gilts. We also hypothesized that Improvest would increase the performance of Duroc–Pietrain-sired gilts enough to reach Duroc-sired untreated gilts levels. Finally, we hypothesized that Improvest-treated gilts, regardless of their genetic line, would reach market weight faster than control gilts and that their carcass would have similar characteristics to those of their respective genetic barrows.

## Materials and Methods

All the animal procedures used in this study were approved by the National Farm Animal Care Council of Canada and follow the Canadian Code of Practice for the Care and Handling of Pigs (Canadian Pork Council, 2014).

### Live Performance

Ten-week-old barrows (physically castrated between 3 and 5 d of age) and gilts (*n* = 846 total pigs) sired by two different genetic lines (DP = Pietrain/Duroc boars; **D** = Duroc boars; sows for both genetic lines were Large White/Landrace) were used in this study. Gilts (*n* = 456) were divided by genetic line (DP and D), randomized by weight, and assigned to Improvest (**IMP**; Zoetis, Parsippany, NJ, USA) treatment or no Improvest treatment (**CON**), while barrows (*n* = 390) were only divided by genetic line (DP and D). Pigs were housed in a mechanically ventilated commercial barn in pens with partially slatted concrete floors at a space allowance of ~0.80 m^2^/pig. This resulted in 6 different treatment groups: DP CON gilts (*n* = 6 pens; 19 pigs/pen); DP IMP gilts (*n* = 6 pens; 19 pigs/pen); D CON gilts (*n* = 6 pens; 10 pigs/pen); D IMP gilts (*n* = 6 pens; 19 pigs/pen); DP barrows (*n* = 10 pens; 19-20 pigs/pen); D barrows (*n* = 10 pens; 19-20 pigs/pen).

Improvest-treated gilts received two 2-mL doses of Improvest subcutaneously in the postauricular region of the neck (following product administration guidelines). The first dose was administered on day 25 of the study and the second dose was administered 6 wk later, on day 67 of the study (Table 1) by trained Improvest personnel. A corn and soybean-based pelleted diet was provided in a 4-phase feeding program (Table 1) for all the pigs in an equivalent manner. Feed and water were provided on an ad libitum basis throughout the duration of the study.

**Table 1.** Ingredient composition and calculated nutrient content of finishing diets fed to all treatment groups (as fed-basis)^1^

Ingredient, %	Phase 1	Phase 2	Phase 3	Phase 4
Corn	30.67	34.33	43.92	48.78
Wheat	15.00	15.00	15.00	15.00
Bakery meal	15.00	15.00	15.00	15.00
Soybean meal	18.40	13.20	9.70	6.30
Canola meal	10.00	10.00	10.00	10.00
DDGS	5.00	7.50	2.60	2.20
Fat	2.50	2.00	1.00	0.00
Limestone	1.09	0.86	0.79	0.81
l-Lysine sulfate (70% BioLys)	0.61	0.61	0.59	0.55
Fat mixer	0.50	0.50	0.50	0.50
Salt	0.35	0.35	0.36	0.37
Premix Elarom Mn 50 F750	0.40	0.20	0.20	0.20
GF premix	0.20	0.20	0.00	0.00
l-Threonine (25 kg)	0.12	0.11	0.11	0.09
Methionine (25K)	0.07	0.04	0.07	0.03
Selko POMix 2%	0.04	0.04	0.04	0.04
Choline liquid	0.02	0.02	0.00	0.00
Trace mineral premix	0.02	0.02	0.02	0.02
Phytase	0.01	0.01	0.01	0.01
Finisher PX	0.00	0.00	0.10	0.10
Calculated analysis, unit				
Dry matter, %	88.48	88.36	88.01	87.80
Fat, %	6.55	6.32	5.07	4.15
Protein, %	20.20	18.75	16.42	15.04
Crude fiber, %	3.71	3.74	3.41	3.33
Neutral detergent fiber, %	10.59	11.11	10.21	10.20
Calcium, %	0.60	0.50	0.45	0.44
Phosphorus, %	0.45	0.44	0.41	0.39
Phytase, P (Inositol), %	0.27	0.25	0.25	0.24
Phytase, FTU	500.0	500.0	500.0	500.0
Sodium, %	0.19	0.19	0.19	0.20
Zinc, total, mg/kg	131.16	130.57	126.83	125.82
Copper, mg/kg	133.0	133.0	118.0	118.0
Selenium, mg/kg	0.30	0.30	0.30	0.30
Vit A, IU/kg	3000.0	3000.0	2000.0	2000.0
Vit D, IU/kg	1000.0	1000.0	400.0	400.0
Vit E, IU/kg	35.0	35.0	35.0	35.0
Choline, mg/kg	121.5	121.5	0.0	0.0
Standardized ileal digestible amino acids				
Lysine, %	1.10	0.99	0.88	0.78
Methionine, %	0.35	0.31	0.31	0.26
Threonine, %	0.70	0.63	0.57	0.51
Tryptophan, %	0.20	0.17	0.15	0.14
Valine, %	0.78	0.71	0.63	0.57
Calculated NEg, kcal/kg	2482.2	2496.4	2501.3	2482.3
Linoleic acid, %	1.83	1.91	1.61	1.48

^1^Phase 1 was fed from ~45 to 59 kg body weight; Phase 2 was fed from 59 to 81 kg body weight; Phase 3 was fed from 81 to 95 kg body weight; Phase 4 was fed from 95 kg to market.

All pigs were initially weighed on day 0 of the study (Table 2). Subsequently, body weight (**BW**) and feed disappearance were measured on days 21, 42, 63, and 84 of the study. All pigs were weighed prior to the first marketing event, with pigs selected for each marketing weighed on the day of marketing (i.e., when they reached 133 ± 2.5 kg). In total, there were four marketing events (Table 2). These measurements (i.e., the differences for BW and feed disappearance observed during each duration) were used to calculate ADG, ADFI, and G:F.

**Table 2.** Timeline of events including day of the study, age of pig, and time from second dose of Improvest

Study day	Age, d	Event	Days post-second dose of Improvest
0	70	Start of study; BW	
21	91	BW	
25	95	First dose of Improvest	
42	112	BW	
63	133	BW	
67	137	Second dose of Improvest	0
84	154	BW	17
119	189	BW for pigs sold; Market event 1^*^	52
126	196	BW for pigs sold; Market event 2^*^	59
133	203	BW for pigs sold; Market event 3^*^	66
140	210	BW for pigs sold; Market event 4^*^	73

Live body weight (BW) was obtained on most study days listed below.

^*^Pigs were marketed when they reached 133 ± 2.5 kg.

### Carcass Characteristics

Pigs were slaughtered under the supervision of the Canadian Food Inspection Agency (CFIA) at a federally inspected processing facility per standard operating procedures. Prior to slaughter, a live weight for each pig was obtained. Hot carcass weight (head-on) and optical grading probe measurements were collected during slaughter. Grading probe measurements (backfat thickness and loin muscle depth) were collected on-line by experienced operators using a Destron PG-100 probe (International Destron Technologies). The handheld grading probe was inserted perpendicularly at the grading site between the third and fourth last ribs, 7 cm off the split line according to Canadian grading standards (Pomar and Marcoux, 2003). Grading probe backfat thickness and loin muscle depth measurements were used to obtain the predicted lean yield value for each carcass using the following equation developed from the 1992 Canadian National Cut-Out study (Canadian Pork Council, 1994):

CLY (%) = 68.1863 − (0.7833 × fat depth) + (0.0689 × lean depth) + (0.0080 × fat depth^2^) − (0.0002 × lean depth^2^) + (0.0006 × fat depth × lean depth)

where CLY is the Canadian Lean Yield value for each carcass (referred to as predicted lean yield herein and throughout), and fat depth and lean depth respectively are the grading probe backfat thickness (mm) and the loin depth (mm) measurements collected at the grading site, respectively, for each carcass.

Carcasses from each marketing event were selected for the carcass cutting tests and meat quality evaluations (Table 3). These evaluations were performed on the left side of the carcasses. Upon the arrival of the carcass sides at the research center, carcasses were separated into primal pieces (401 ham, 403 whole shoulders, 409 bellies, and 410 loins) according to North American Institutional Meat Purchase Specifications (NAMP, 2006; IMPS, 2014) guidelines using a bandsaw and weighed as skin-on, bone-in, untrimmed pieces.

**Table 3.** The number (and percentage) of Duroc–Pietrain-sired (DP) and Duroc-sired (D) pigs marketed and evaluated for carcass cut out and meat quality at each marketing event; Barrows were physically castrated and Improvest (IMP) was administered to half the gilts.

Genetic sire line	Sex	IMP	Marketing event 1		Marketing event 2		Marketing event 3		Marketing event 4		
			Pigs sold	Carcass cutouts	Pigs sold	Carcass cutouts	Pigs sold	Carcass cutouts	Pigs sold	Carcass cutouts	Total
DP	Gilt	No	18	5 (27.8%)	18	4 (22.2%)	45	14 (31.1%)	28	22 (78.6%)	45/109 (41.2%)
DP	Gilt	Yes	39	16 (41.0%)	31	21 (67.7%)	33	10 (30.3%)	10	6 (60.0%)	53/113 (46.9%)
D	Gilt	No	25	6 (24.0%)	26	8 (30.8%)	38	11 (28.9%)	12	8 (66.7%)	33/101 (32.7%)
D	Gilt	Yes	36	11 (30.6%)	35	11 (31.4%)	29	7 (24.1%)	9	8 (88.9%)	37/109 (33.9%)
DP	Barrow	No	44	12 (27.3%)	48	13 (27.1%)	75	24 (32.0%)	24	8 (33.3%)	57/191 (29.8%)
D	Barrow	No	52	23 (44.2%)	46	18 (39.1%)	70	9 (12.9%)	23	8 (34.8%)	58/191 (30.4%)
		SUM	214	73	204	75	290	75	106	60	283/814 (34.8%)

The loin was further fabricated by removing a 3-cm section at the Canadian grading site (i.e., between the third and fourth last rib). The anterior surface area of the loin (i.e., loin eye area) was traced using transparent vellum paper and a marker and then quantified using a planimeter. Meat quality measurements were also collected on the anterior face of this loin section. These measurements included Japanese color score, NPPC marbling score, instrumental color, pH, and drip loss. Japanese color score and NPPC marbling score were evaluated by trained personnel and recorded to the nearest half number. Instrumental color was measured with a calibrated, handheld Minolta CR-400 Chroma meter (Konica Minolta Sensing Americas, Inc, Ramsey, NJ, USA) with illuminant D_65_ and 0° viewing angle settings. Each measurement by the Chroma meter was reported using the L*, a*, and b* color space. pH was measured 24 h postmortem using a calibrated pH meter (Orion 4; Thermo Scientific; Waltham, MA, USA). Drip loss was measured over a 48-h period with the EZ cup method described previously by Rassmussen & Andersson (1996).

### Statistical Analyses

This trial was conducted as a completely randomized design with six treatment groups and a single treatment being allotted to each pen of pigs. Pen was therefore considered the experimental unit for this study.

Data for variables collected over time on the same pig including BW, ADG, ADFI, and G:F were analyzed by a linear mixed model approach for repeated measures. Using the Proc Mixed Procedure (SAS 9.4, Cary, NC), these variables were analyzed with a model that considered the fixed effects of treatment, day, and the interaction of treatment-by-day and the random effects of pen(treatment) and the residual error. Day was the repeated factor and pen was the subject. Initial weights were used as a covariate in the analysis. BW, ADG, ADFI, and G:F were also analyzed by Improvest treatment and genetic line with a model that considered the fixed effects of treatment, day, genotype, and all interactions and the random effect of pen(treatment). Day was the repeated factor and pen was the subject.

The covariance structure in the repeated measures analysis was investigated using five structural assumptions, namely, compound symmetry, power [SP(POW)], first-order autoregressive [AR(1)], heterogeneous first-order autoregressive [ARH(1)], and unstructured. The assumption giving the minimum value of Akaike’s Information Criterion was selected in the final analysis. Treatment Least Square Means (**LSMeans**) were calculated for each group. Comparisons of LSMeans were performed by the two-sided Student’s t-test at the 5% level of significance. Treatment and treatment-by-day and/or treatment-by-genetic line were assessed at the 5% level.

Data for variables collected as a single timepoint such as initial weight, total ADG, total ADFI, total G:F, days to market, and continuous carcass variables were analyzed by a linear mixed model approach (LMM). Using the Proc Mixed Procedure these variables were analyzed with a model that considered initial weight as the covariate, the fixed effect of treatment and the random effects of pen-within-treatment, and the residual error. BW, ADG, ADFI, G:F, and carcass variables were also analyzed by Improvest treatment and genetic line with a model that considered the fixed effects of treatment, day, genetic line, and all interactions and the random effect of pen(treatment). Covariance structures and LSMeans for each group were computed as stated above and compared by the two-sided Student’s *t* test at the 5% level of significance as stated previously. Carcass variables that were scored (i.e., categorical data) were analyzed with a Chi square test by the SAS Proc Freq procedure.

## Results

### Body Weight

At the start of the study, there was a significant difference (*P* < 0.01) in BW where DP barrows were the heaviest (*P* < 0.01) compared to all other treatment groups, while D gilts and barrows were the lightest (*P* < 0.01) and DP gilts were intermediate (Table 4). Thus, starting weight was used as a covariate in the repeated measures analysis, and therefore, no differences in weight were observed on day 21 or day 42 of the study. Just prior to the administration of the second dose of Improvest (i.e., day 63), DP IMP gilts were lighter (*P* ≤ 0.05) than DP barrows, and D IMP gilts were lighter (*P* ≤ 0.05) than D barrows (Table 4). On day 84, DP IMP gilts remained lighter than DP barrows, and DP IMP gilts were lighter than D CON gilts (Table 3). By the time of marketing (i.e., final weight), which was conducted when the pigs reached 133 ± 2.5 kg, there was no difference in weight; however, DP IMP gilts and DP barrows reached market weight sooner (*P* ≤ 0.05) than DP CON gilts (Table 4), which had the most days on feed. The D CON and D IMP gilts had similar days on feed compared to D barrows. This resulted in the DP IMP gilts and DP barrows to have a smaller, and similar, coefficient of variation (i.e., 1.72% and 1.67%) compared to the DP CON (2.41%) gilts, suggesting DP IMP gilts and DP barrows were more uniform in weight compared to DP CON gilts. This relationship was not observed in the D pigs (1.67%, 2.96%, and 3.05% for D CON gilts, D IMP gilts, and D barrow, respectively).

**Table 4.** Pig performance by day in Duroc–Pietrain control gilts (DP CON gilts), Duroc–Pietrain Improvest gilts (DP IMP gilts), Duroc control gilts (D CON gilts), Duroc Improvest gilts (D IMP gilts), Duroc–Pietrain barrows (DP barrows), and Duroc barrows (D barrows)

	Treatment*							*P* value		
Day	DP CON gilts	DP IMP gilts	D CON gilts	D IMP gilts	DP barrows	D barrows	SEM	Trt	Day	Trt*dayv
Live weight, kg										
0	30.71^b^	30.79^b^	29.37^c^	29.37^c^	31.28^a^	29.30^c^	0.05	<0.01	—	—
21	47.53	48.05	49.47	49.24	47.49	49.63	1.26	0.24	<0.01	< 0.01
42	66.41	65.86	68.18	67.11	66.85	68.44	1.30			
63	87.69^abc^	86.40^bc^	89.66^abc^	87.89^ac^	89.55^ad^	89.89^bd^	1.37			
84	110.12^ab^	108.43^b^	114.15^a^	111.79^ab^	111.81^a^	112.30^ab^	1.50			
Final	135.09	136.78	137.19	136.88	136.53	135.23	1.33			
Days on feed	114.29^a^	109.86^bc^	111.30^bc^	109.29^c^	111.82^b^	110.98^bc^	0.83	<0.01	—	—
Average daily gain, kg/d										
0 to final	0.91^c^	0.97^ab^	0.97^ab^	0.99^a^	0.94^bc^	0.96^ab^	0.02	0.06		
0 to 21	0.80	0.84	0.81	0.83	0.84	0.84	0.04	0.34	<0.01	< 0.01
21 to 42	0.89	0.84	0.87	0.85	0.91	0.90	0.05			
42 to 63	1.01^ab^	0.97^b^	1.02^ab^	0.99^ab^	1.07^a^	1.02^ab^	0.05			
63 to 84	1.07^ab^	1.03^b^	1.17^a^	1.12^ab^	1.04^ab^	1.08^b^	0.05			
84 to Final	0.81^c^	1.09^ab^	0.95^bc^	1.12^a^	0.83^c^	0.97^bc^	0.07			
Average daily feed intake, kg/d										
0 to final	2.38	2.48	2.50	2.53	2.51	2.51	0.04	0.12		
0 to 21	1.42^a^	1.43^ab^	1.58^ab^	1.52^b^	1.46^ab^	1.61^a^	0.08	0.07	<0.01	< 0.01
21 to 42	1.94^abc^	1.87^c^	2.02^bc^	1.96^bc^	2.00^ab^	2.12^a^	0.08			
42 to 63	2.34^cd^	2.24^d^	2.62^ab^	2.53^bc^	2.61^ab^	2.78^a^	0.10			
63 to 84	2.84^b^	2.87^ab^	3.09^ab^	3.02^ab^	2.87^ab^	3.11^a^	0.09			
84 to final	2.92^c^	3.52^ab^	3.32^b^	3.77^a^	2.92^c^	3.21^bc^	0.12			
Feed efficiency, G:F, kg/kg										
0 to final	0.38	0.39	0.38	0.38	0.37	0.38	0.01	0.12		
0 to 21	0.55^abcde^	0.57^acd^	0.53^cde^	0.57^ab^	0.54^be^	0.54^cde^	0.01	<0.01	<0.01	<0.01
21 to 42	0.45	0.44	0.45	0.45	0.44	0.44	0.01			
42 to 63	0.43^ab^	0.43^a^	0.40^ab^	0.40^ab^	0.40^bc^	0.38^c^	0.01			
63 to 84	0.37^ab^	0.36^ab^	0.38^a^	0.38^a^	0.36^ab^	0.35^b^	0.01			
84 to final	0.28^b^	0.31^a^	0.29^ab^	0.30^ab^	0.28^b^	0.31^ab^	0.01			

Weight 0 was included as a covariate in the repeated measures analysis.

*Paternal genetic line described; Maternal line for all treatments was Large White-Landrace.

^abcde^LSMeans ± SEM within a row with different superscripts differ; *P* ≤ 0.05.

### Average Daily Gain

There was a significant (*P* < 0.01) treatment-by-day interaction for ADG from day 0 to the final day of the study (Table 4). ADG was similar across treatments until days 42 to 63, where DP barrows had greater (*P* ≤ 0.05) ADG compared to DP IMP gilts with all other treatments being intermediate. From days 63 to 84 D CON gilts had a greater ADG (*P* ≤ 0.05) compared to D barrows and DP IMP gilts with all other groups being intermediate. Prior to marketing (days 84 to final), DP IMP gilts had a greater ADG (*P* ≤ 0.05) than the DP CON gilts and DP barrows, which were similar. Moreover, D IMP gilts had a greater ADG (*P* ≤ 0.05) than the D CON gilts and D barrows, which were similar (Table 3). There was a tendency (*P* = 0.06) for a treatment effect on overall ADG from day 0 to market with DP IMP gilts being similar to DP barrows with both having a greater (unprotected *F* test; *P* ≤ 0.05) ADG than D CON gilts, while D CON gilts, D IMP gilts, and D barrows performed similarly throughout the study (Table 4).

### Average Daily Feed Intake

While there was no difference when ADFI was calculated from day 0 to market (*P* = 0.12), there were differences at various timepoints throughout the study. From days 0 to 21, there were no differences between the DP CON gilts, DP IMP gilts, or DP barrows. However, D barrows had a greater ADFI (*P* ≤ 0.05) compared to DP IMP gilts, with D CON gilts being intermediate. From days 21 to 42, DP barrows consumed more feed compared to DP IMP gilts, with DP CON gilts being intermediate, whereas D barrows consumed more (*P* ≤ 0.05) feed compared to D CON gilts and D IMP gilts which did not differ (Table 4). While DP barrows consumed more (*P* ≤ 0.05) feed from days 42 to 63 compared to the DP CON and DP IMP gilts, which did not differ, D barrows consumed more (*P* ≤ 0.05) feed compared to DP IMP gilts with DP CON gilts being intermediate. ADFI was similar within a genotype from days 63 to 84, the only difference being D barrows consuming more (*P* ≤ 0.05) than DP CON gilts. From day 84 to final, DP IMP gilts had a greater (*P* ≤ 0.05) ADFI compared to both DP CON gilts and DP barrows, which did not differ; likewise, D IMP gilts had a greater (*P* ≤ 0.05) ADFI compared to both D CON gilts and D barrows, which did not differ (Table 4).

### Feed Efficiency

While there were no difference for G:F from day 0 to market (*P* = 0.12), there were differences at various timepoints throughout the study. From days 0 to 21, the DP barrows had a lower (*P* ≤ 0.05) G:F compared to DP IMP gilts, with DP CON gilts being intermediate. The G:F ratio was increased (*P* ≤ 0.05) in D IMP gilts compared to D CON gilts and D barrows, which did not differ (Table 4). The G:F ratio was similar from days 21 to 42, however from days 42 to 63, the barrows of each genotype had a lower (*P* ≤ 0.05) G:F ratio compared to their respective gilt counterparts, which did not differ. While there was no difference in G:F within the DP genotype, the D barrows had a reduced (*P* ≤ 0.05) G:F ratio compared to the D CON gilts and D IMP gilts, which did not differ. Lastly, from day 84 to market, the G:F ratio was increased (*P* ≤ 0.05) in DP IMP gilts compared to both DP CON gilts and DP barrows, which did not differ; whereas there were no differences within the D genotype (Table 4).

### Carcass Measurements

At the plant, live weight was greater (*P* ≤ 0.05) for DP IMP gilts and DP barrows compared to DP CON gilts. There was no difference in live weight within the D genotype. D CON gilts were heavier (*P* ≤ 0.05) than DP CON gilts (Table 5). Hot carcass weights were greater (*P* ≤ 0.05) in DP barrows compared to DP CON gilts with DP IMP gilts being intermediate. The D barrows had a heavier (*P* ≤ 0.05) hot carcass weight compared to the D CON gilts and D IMP gilts, which did not differ. While there was no difference within the DP pigs for dressing percentage, D barrows had a greater (*P* ≤ 0.05) dressing percentage than the D CON gilts and D IMP gilts, which did not differ from one another (Table 5). There was no difference in muscle depth, but backfat thickness was increased in IMP versus CON gilts within their respective genotypes, with IMP gilts having similar backfat thickness as their barrow counterparts. Upon calculation of CLY, predicted lean yield values were reduced (*P* ≤ 0.05) in DP IMP gilts and DP barrows compared to DP CON gilts. Likewise, CLY predicted lean yield values were reduced (*P* ≤ 0.05) in D IMP gilts and D barrows compared to D CON gilts.

**Table 5.** Carcass data in Duroc–Pietrain control gilts (DP CON gilts), Duroc–Pietrain Improvest gilts (DP IMP gilts), Duroc control gilts (D CON gilts), Duroc Improvest gilts (D IMP gilts), Duroc–Pietrain barrows (DP barrows), and Duroc barrows (D barrows)

	Treatment^*^						SEM	*P* value
Variable	DP CON gilts	DP IMP gilts	D CON gilts	D IMP gilts	DP barrows	D barrows		
Live Wt, kg	131.64^b^	133.43^a^	133.10^a^	132.19^ab^	133.41^a^	133.46^a^	0.54	0.02
HCW, kg	105.88^b^	106.95^ab^	106.00^b^	105.75^b^	107.64^a^	107.91^a^	0.60	0.01
Dressing percentage	80.46^abc^	80.13^abc^	79.68^c^	80.05^bc^	80.71^ab^	80.86^a^	0.32	0.04
Muscle depth, mm	71.14	70.74	71.85	72.08	70.68	70.59	1.01	0.75
Backfat thickness, mm	14.60^c^	16.56^b^	13.74^c^	16.63^ab^	16.97^ab^	17.90^a^	0.56	<0.001
CLY, %	63.03^a^	62.05^b^	63.51^a^	62.08^bc^	61.85^bc^	61.41^c^	0.27	<0.001
Loin surface area, cm^2^	57.17^a^	56.05^abc^	57.83^a^	56.46^ab^	54.80^bc^	54.24^c^	0.91	0.01
Ham (IMPS #401), kg	12.48	12.51	12.58	12.50	12.29	12.41	0.09	0.18
Shoulder (IMPS #403), kg	12.43^b^	12.53^b^	12.60^b^	12.31^b^	12.58^b^	12.90^a^	0.11	<0.01
Loin (IMPS #410), kg	12.63^c^	13.23^a^	12.79^bc^	13.07^ab^	13.17^ab^	13.10^ab^	0.17	0.04
Belly (IMPS #409), kg	8.52	8.37	8.33	8.24	8.54	8.38	0.10	0.16
pH 24	5.71	5.70	5.72	5.70	5.76	5.72	0.02	0.23
Minolta L^*^	51.88^b^	52.80^ab^	52.82^ab^	53.88^a^	52.18^b^	53.12^ab^	0.56	0.08
Minolta a^*^	3.85	3.61	3.87	4.14	4.02	4.06	0.26	0.55
Minolta b^*^	9.82	9.48	9.97	10.23	9.93	9.97	0.24	0.19
Drip loss, %	4.17^ab^	3.10^b^	4.66^a^	4.99^a^	4.07^ab^	4.03^ab^	0.53	0.06

^*^Paternal genetic line is described.

Maternal line for all treatments were Large White-Landrace.

CLY = Canadian Lean Yield; see text for equation.

^abc^LSMeans ± SEM within a row with different superscripts differ; *P* ≤ 0.05.

There were no differences in treatment on ham or belly weights. There was, however, an increase (*P* ≤ 0.05) for shoulder weights in D barrows compared to all other treatments, which did not differ from one another (Table 5). Loins were heavier (*P* ≤ 0.05) in DP IMP gilts compared to DP CON gilts, with DP IMP gilts and DP barrows being similar in weight. There was no difference in loin weight for D genotype pigs (i.e., D CON gilts, D IMP gilts, or D barrows). The loin surface area was greater in DP CON and D CON gilts compared to DP and D barrows, with DP IMP and D IMP gilts being intermediate. While pH, Minolta a* and b* were similar across treatment, there was a tendency (*P* = 0.08) for Minolta L* to be reduced (*P* ≤ 0.05) in DP CON gilts versus DP barrows with DP IMP gilts being intermediate (Table 5). Drip loss was similar across genotype groups, but DP IMP gilts had reduced (*P* ≤ 0.05) drip loss compared to D IMP gilts (Table 5).

**Figure 1.**
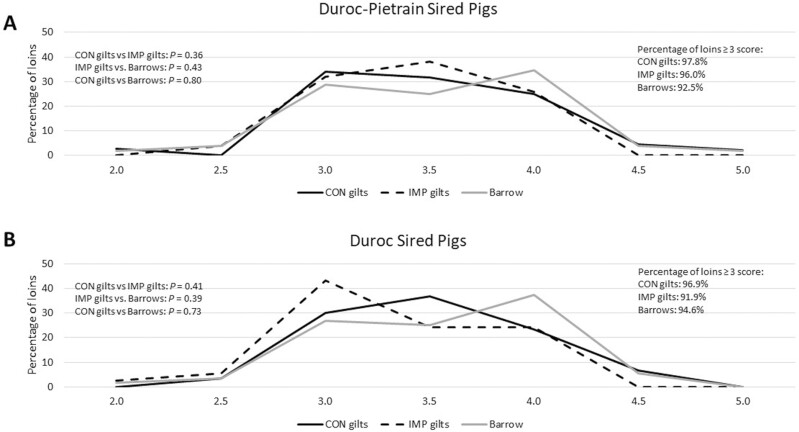
Japanese Color Score of loins evaluated in Duroc–Pietrain-sired pigs (A) and Duroc-sired pigs (B). Chi square analyses were performed for differences between treatment groups within a genotype (left side of graph). The percentage of loins that met the preferred color score of 3 or greater is presented on the right side of the slide.

**Figure 2.**
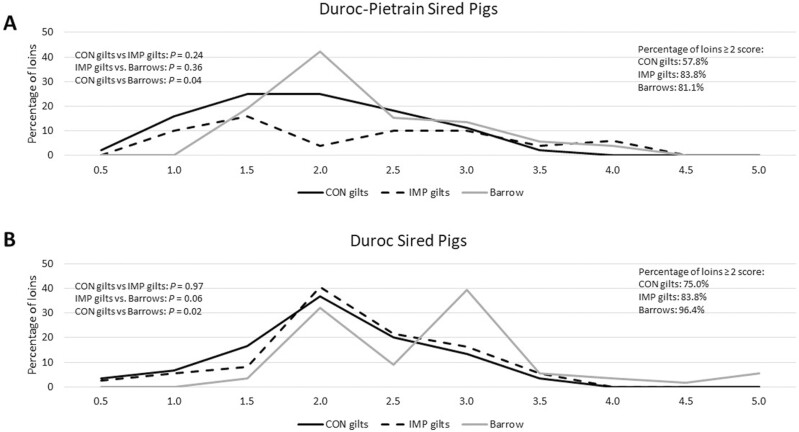
Visual Marbling Score of loins evaluated in Duroc–Pietrain-sired pigs (A) and Duroc-sired pigs (B). Chi square analyses were performed for differences between treatment groups within a genotype (left side of graph). The percentage of loins that met the preferred color score of 3 or greater is presented on the right side of the slide.

The categorical data for loin Japanese color score and loin marbling were assessed with Chi square analysis, and while there were no differences (*P* ≥ 0.35) on pattern profiles within a genotype for Japanese color score (Figure 1), there were pattern differences within marbling (Figure 2). While DP CON and DP IMP gilts were similar in their marbling score pattern, DP IMP gilts were also similar to DP barrows, indicating that DP barrows and DP IMP gilts showed a similar distribution of marbling (Figure 2A). There was a difference between DP barrows and DP CON gilts (*P* = 0.04), with DP barrows having a shift of increased marbling scores compared to the DP CON gilts. In the D genotype, while there was no difference in marbling between D CON gilts and D IMP gilts, there was a tendency (*P* = 0.06) and a statistical difference (*P* = 0.02) for D barrows to have more marbling than D IMP gilts and D CON gilts, respectively (Figure 2B). When the percentage of ideal scores was evaluated (i.e., a color score ≥ 3 and marbling score ≥ 2) and combined for an ideal loin, the Duroc barrows had the greatest percentage (data not analyzed). It is of interest that within a genetic sire line, the gilts that received Improvest had an increased percentage of ideal loins compared to the gilts that did not receive Improvest (Table 6).

**Table 6.** The percentage of loins that had a Japanese color score ≥ 3, a marbling score ≥ 2, and the percentage of loins that had both a ≥ 3 color score and ≥ 2 marbling score within the genetic sire line, sex, and Improvest treatment

Genetic sire line	Sex	Improvest treatment	Color score ≥ 3	Marbling score ≥ 2	Combine pass rate
Duroc–Pietrain	Gilt	No	97.8%	57.8%	57.8%
Duroc–Pietrain	Gilt	Yes	96.0%	83.8%	70.0%
Duroc–Pietrain	Barrow	No	92.5%	81.1%	79.2%
Duroc	Gilt	No	96.9%	75.0%	75.0%
Duroc	Gilt	Yes	91.9%	83.8%	78.4%
Duroc	Barrow	No	94.6%	96.4%	96.4%

## Discussion

We accept our hypothesis that Improvest increases ADG and ADFI in gilts. Increases of 18% in ADG and ADFI have been reported in gilts upon the administration of a second dose of Improvest (i.e., during the time period post-second dose) (Oliver et al., 2003; Van den Broeke et al., 2016; Rodrigues et al., 2019; Nautrup et al., 2020). In this study, it was not possible to evaluate the specific effects of Improvest on ADG and ADFI immediately after the second dose since BW was not recorded at the time of injection. The second dose of Improvest was administered on day 67 of the study, while BW and feed intake data were collected on day 84 and in the final day of the study as pigs reached market weight. Since it has also been reported that Improvest modifies feed intake in female pigs after ~5 to 9 d post-second dose (Van den Broeke et al., 2016; Nautrup et al., 2018, 2020), it is reasonable to describe Improvest effects in production traits after this period. Therefore, in this study, we report Improvest effects on production overall and from day 84 to the final day of the study. There was a 25% and 17% increase in ADG and ADFI, respectively, after the administration of a second dose of Improvest in gilts. This data agrees with previous studies which report 10% to 30% increases in ADG and 14% to 22% increases in ADFI post-second dose (Oliver et al., 2003; Van den Broeke et al., 2016; Rodriguez et al., 2018; Nautrup et al., 2020). Improvements in ADG post-second dose are primarily due to the increase in ADFI, but there may be some redirection of nutrients away from reproductive tract development and towards lean/fat deposition (Van den Broeke et al., 2016; Allison et al., 2021).

We also report no differences in feed efficiency after the second dose of Improvest in gilts, which is similar to previously reported work (Oliver et al., 2003; Nautrup et al., 2020); however, others have reported significant increases in G:F (Bohrer et al., 2014; Van den Broeke et al., 2016), or a slight decrease in G:F (Rodriguez et al., 2018). We believe the variety of responses might be due to the different genetic lines. Indeed, in this study we report greater performance responses to Improvest after the second dose in Duroc–Pietrain-sired gilts compared with Duroc-sired gilts. It is important to highlight that the Duroc–Pietrain terminal sire line was the underperforming genetic line previous to a second dose of Improvest. Decreased growth performance has been reported in Pietrain-sired pigs compared to Duroc-sired pigs (Augspurger et al., 2002; Edwards et al., 2006; Morales et al., 2013). In this study, we observed a 34% and 21% increase in ADG and ADFI after the second dose of Improvest is administered in Duroc–Pietrain-sired gilts, and 18% and 14% increases in the same variables for Improvest-treated Duroc-sired gilts. Moreover, feed efficiency after the second dose of Improvest increased by 10% in Improvest-treated Duroc–Pietrain-sired gilts compared to CON gilts of the same genetic line, whereas it did not improve in Improvest-treated or untreated Duroc-sired gilts. Similar results have been reported in barrows in which greater ADG and ADFI along with an increased feed efficiency enabled Improvest-treated Pietrain-sired barrows to reach the level of performance of control Duroc-sired barrows (Morales et al., 2013). Our data agrees with previously published data that Improvest increases ADG and ADFI in gilts after the second dose is administered, in both Duroc–Pietrain and Duroc-sired gilts.

As this project had a targeted final market weight rather than a fixed-time ending, days to market weight was analyzed to further assess Improvest effects on live weights. Days to market was decreased in Duroc–Pietrain IMP gilts compared to their nontreated female counterparts. Improvest-treated gilts were similar to physically castrated barrows within their genetic line. In this study, ADG was reduced in the Improvest-treated gilts compared to other groups before day 84 of the study. However, we observed a significant increase in ADG (secondary to an increase in ADFI) from day 84 to the final day of the study in Improvest-treated gilts. This resulted in fewer days to reach market weight. Moreover, this positive effect of ADG in reducing days to market after day 84 was amplified in control Duroc–Pietrain-sired gilts, which required the longest time to reach market weight. Furthermore, both Improvest-treated groups had the greatest percentage of pigs sold in the first marketing event and the fewest in the last marketing event. On the contrary, Duroc–Pietrain control gilts had the lowest percentage dispatched in the first marketing event and the greatest on the last compared to all the other groups. A uniform final weight distribution is desired by producers because it enables consistent planning for marketing and could decrease the number of marketing dates. In addition, a uniform carcass weight is desired by packers and processors as it improves efficiency within the plant and size/weight consistency in the merchandized products.

In this study, as all pigs were marketed to the same live BW, it was not surprising that HCW did not greatly differ among treatment groups (2.16 kg range between all treatment groups), and frankly, this parameter should be interpreted with caution due to the marketing strategy implemented. There was an increase in backfat (~2.4 mm) and loin weight (~0.44 kg) for Improvest-treated gilts compared to untreated gilts, which is similar in magnitude to values reported between untreated gilts and barrows in this study. In general, several traits that barrow carcasses exhibit are preferred to that of gilt carcasses, in particular when fat deposition and fat quality are sorting criteria for merchandized products such as loins and bellies (Boler et al., 2014; Kyle et al., 2014). All the while, Pietrain-sired pigs tend to have less backfat and more lean muscle percentage compared to Duroc-sired pigs, making the comparison of the two different genetic groups of pigs in this study of interest (Kušec et al., 2004; Morales et al., 2013; Redifer et al., 2020). In our study, barrows from both genetic lines showed the greatest amount of backfat, while untreated (i.e., CON) gilts from both genetic lines showed the lowest amount of backfat. Similar results between barrows and gilts have been widely documented (Boler et al., 2014; Kyle et al., 2014; Nautrup et al., 2018, 2020). In this study, Improvest-treated gilts from both genetic lines showed an improvement in backfat, with Duroc-sired gilts reaching barrow level backfat and Duroc–Pietrain-sired gilts being intermediate.

Meat quality parameters in this study were presented with two different approaches. Objective measurements (pH, instrumental color, and drip loss) were presented as LSMeans (Table 4), while subjective measurements (Japanese color score and visual marbling score) were presented as distributions (Table 5, Figures 1 and 2). With the exception of drip loss (where slightly less drip loss was observed for the Duroc–Pietrain-sired Improvest gilts compared with the Duroc-sired Improvest gilts and Duroc-sired control gilts), objective measurements were similar among the treatments. For subjective measurements of quality, clear and meaningful differences were observed between the different fixed effects. Overall, loins from Duroc-sired pigs had greater levels of loin pass rate (defined as Japanese color score ≥ 3.0 and marbling ≥ 2.0) compared with Duroc–Pietrain-sired pigs, loins from barrows had greater levels of loin pass rate compared with gilts, and Improvest-treated gilts had greater levels of loin pass rate compared with untreated gilts. Similar findings have been reported by previous researchers; for instance, both Lowell et al. (2019) and Redifer et al. (2020) reported greater visual color and visual marbling scores in Duroc-sired pigs versus Pietrain-sired pigs and in barrows versus gilts. The improvements in loin primal pass rate observed between Improvest-treated gilts versus nontreated gilts were primarily driven by improvements in subjective marbling score, a finding that has been previously reported by multiple studies and is driven by the increased levels of fat deposition associated with the suppression of estrus and ovarian activity (Nautrup et al., 2020).

This study builds the body of evidence that Improvest increases ADG and ADFI in gilts after a second dose is administered. We also suggest that Improvest may have a greater beneficial impact on performance characteristics via feed efficiency in Duroc–Pietrain-sired gilts. Furthermore, we suggest that the benefits elicited by Improvest in these traits could assist producers who seek greater uniformity in marketing weights. Finally, Improvest use in gilts partially increases desired carcass characteristics and meat quality, shifting several traits closer to that of barrows.
